# Application of Multi-Scale Fusion Attention U-Net to Segment the Thyroid Gland on Localized Computed Tomography Images for Radiotherapy

**DOI:** 10.3389/fonc.2022.844052

**Published:** 2022-05-26

**Authors:** Xiaobo Wen, Biao Zhao, Meifang Yuan, Jinzhi Li, Mengzhen Sun, Lishuang Ma, Chaoxi Sun, Yi Yang

**Affiliations:** ^1^ Department of Radiotherapy, Yunnan Cancer Hospital, Kunming, China; ^2^ Department of Neurosurgery, Yunnan Cancer Hospital, Kunming, China

**Keywords:** U-Net model, multi-scale fusions, medical-image segmentation, thyroid, radiotherapy

## Abstract

**Objective:**

To explore the performance of Multi-scale Fusion Attention U-Net (MSFA-U-Net) in thyroid gland segmentation on localized computed tomography (CT) images for radiotherapy.

**Methods:**

We selected localized radiotherapeutic CT images from 80 patients with breast cancer or head and neck tumors; label images were manually delineated by experienced radiologists. The data set was randomly divided into the training set (n = 60), the validation set (n = 10), and the test set (n = 10). We expanded the data in the training set and evaluated the performance of the MSFA-U-Net model using the evaluation indices Dice similarity coefficient (DSC), Jaccard similarity coefficient (JSC), positive predictive value (PPV), sensitivity (SE), and Hausdorff distance (HD).

**Results:**

For the MSFA-U-Net model, the DSC, JSC, PPV, SE, and HD values of the segmented thyroid gland in the test set were 0.90 ± 0.09, 0.82± 0.11, 0.91 ± 0.09, 0.90 ± 0.11, and 2.39 ± 0.54, respectively. Compared with U-Net, HRNet, and Attention U-Net, MSFA-U-Net increased DSC by 0.04, 0.06, and 0.04, respectively; increased JSC by 0.05, 0.08, and 0.04, respectively; increased SE by 0.04, 0.11, and 0.09, respectively; and reduced HD by 0.21, 0.20, and 0.06, respectively. The test set image results showed that the thyroid edges segmented by the MSFA-U-Net model were closer to the standard thyroid edges delineated by the experts than were those segmented by the other three models. Moreover, the edges were smoother, over–anti-noise interference was stronger, and oversegmentation and undersegmentation were reduced.

**Conclusion:**

The MSFA-U-Net model could meet basic clinical requirements and improve the efficiency of physicians’ clinical work.

## Introduction

Head and neck tumors and breast cancer are currently the tumors with relatively higher morbidity and mortality rates worldwide (1). In 2020, 19.29 million new cancer cases occurred worldwide, of which 4.57 million (23.7%) were in China. Radiotherapy is an effective and common method for treating head and neck cancer and breast cancer (2–4). Accurately delineating organs at risk (OARs) when designing radiotherapy plans can effectively avoid radiation side effects. At present, physicians are responsible for outlining OARs, making the process subjective, time consuming, and labor intensive.

The rapid development of artificial intelligence (AI) enabled Ronneberger et al. (5) to propose the U-Net neural network model in 2015. The delineation method based on deep learning (DL) has gradually been developed and applied in clinical work (6–10). Ye et al. (7) used an improved model, a dense-connectivity embedding U-Net, to train and segment the T1 and T2 magnetic resonance imaging (MRI) images of 44 patients with nasopharyngeal carcinoma; the authors obtained a Dice similarity coefficient (DSC) of 0.87 after tenfold cross-validation. Automatized delineation of the thyroid gland on localized CT images for radiotherapy has been critical in radiotherapy planning (11). Zhai et al. (12) found that the patients who received thyroid mean radio dose of ≥45 Gy had a 4.9 times increased risk of hypothyroidism than those with lower mean radio dose. Akın et al. (13) conducted a retrospective study on 122 patients who received three-dimensional conformal radiation therapy (3D-CRT) for breast cancer. They found that ①functional abnormalities occurred in the thyroid gland which was exposed to total radiation doses of 26 to 30 Gy; ②44% of the patients were exposed to a radiation dose of >26 Gy. Other studies showed that 2 years after patients with head and neck tumors received radiotherapy, their incidence of hypothyroidism was 36%; moreover, this incidence increased along with follow-up time (14, 15). Therefore, in radiotherapeutic planning, radiation must be limited to the thyroid gland. Narayanan D. et al. (16) used multi-atlas label fusion (MALF) and random forest (RF) to automatically segment the thyroid gland on CT and found that MALF with RF presented better segmentation with the DSC being 0.76 ± 0.11, which was significantly better than the individual MALF and RF methods. Chang et al. (17) used a progressive learning vector quantization neural network to segment the thyroid on CT and their experimental results showed that the proposed method could effectively segment thyroid glands with its average SE being 88.43%. He et al. (18) used deep convolutional neural network to segment the thyroid gland on noncontrast-enhanced head and neck CTs and found that their proposed method had significantly improved performance. Considering that CT localization for radiotherapy involves a simulated-positioning, large-aperture CT (SOMATOM Sensation Open, 24 rows, Φ85 cm; Siemens Healthcare, Forchheim, Germany), which is limited by small size and poor image resolution, automatic segmentation of the thyroid gland based on a DL model is difficult. The performance of such a model on localized CT images for radiotherapy requires further exploration. In the deep learning study, the combination of HRNet and SE is common (19). In HRNet, multiple parallel networks with different resolutions are used to extract features and multi-scale fusions are repeatedly performed during feature extraction to ensure that the model can fully obtain information of different scales (20). The cSE module enables the model to pay more attention to major channel features and suppresses those minor channel features (21). Therefore, in this study, we proposed a model that combined a Spatial Squeeze and Channel Excitation Block (cSE) attention mechanism with HRNet on the basis of U-Net and used it to segment the thyroid gland on localized CT images to help delineate the gland as an OAR in radiotherapy.

## Materials and Methods

### Data Set Acquisition

**Figure 1:**
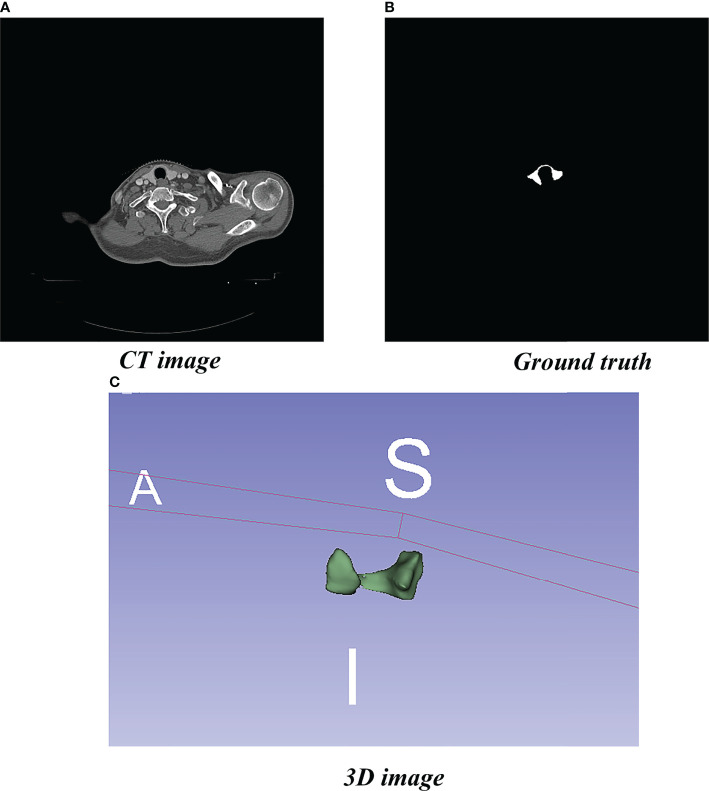
Localized CT image, Ground truth, and 3D image. **(A)** Standard image of the imported model (CT image). **(B)** Corresponding label image (Ground truth). **(C)** Thyroid gland drawn in 3D.

We obtained the experimental data set in this study from 80 patients with nasopharyngeal carcinoma or breast cancer who were admitted to the Department of Radiotherapy of Yunnan Cancer Hospital (Kunming, China) from June 2014 to April 2019. Localization for each patient was simulated using a SOMATOM Sensation Open 24 CT scanner. CT images were obtained in Digital Imaging and Communications in Medicine (DICOM) format with slices being 5 or 3 mm thick and pixels being 512 × 512. Senior radiotherapists drew the label images on the CT images in DICOM, using 3D Slicer software version 4.11. The label images were converted from DICOM to PNG format (**Figure 1**).

We divided the data set (6:1:1) into training, validation, and test sets. Due to the small number of medical data sets and the high cost of drawing, collecting a sufficiently large number of data sets was difficult; however, a training data set that was too small would have created a risk of overfitting the model. To avoid this risk, in this study we expanded the training sample data set size by means of rotation, flipping, zooming, and shearing.

### Data Set Preprocessing

To better highlight the region of interest (ROI), we first converted CT image pixels into Hounsfield unit (HU) values and then adjusted the window width and level of the converted data to highlight the thyroid gland. Finally, we used adaptive histogram equalization to further enhance the contrast and normalize the images.

### Model Framework

**Figure 2:**
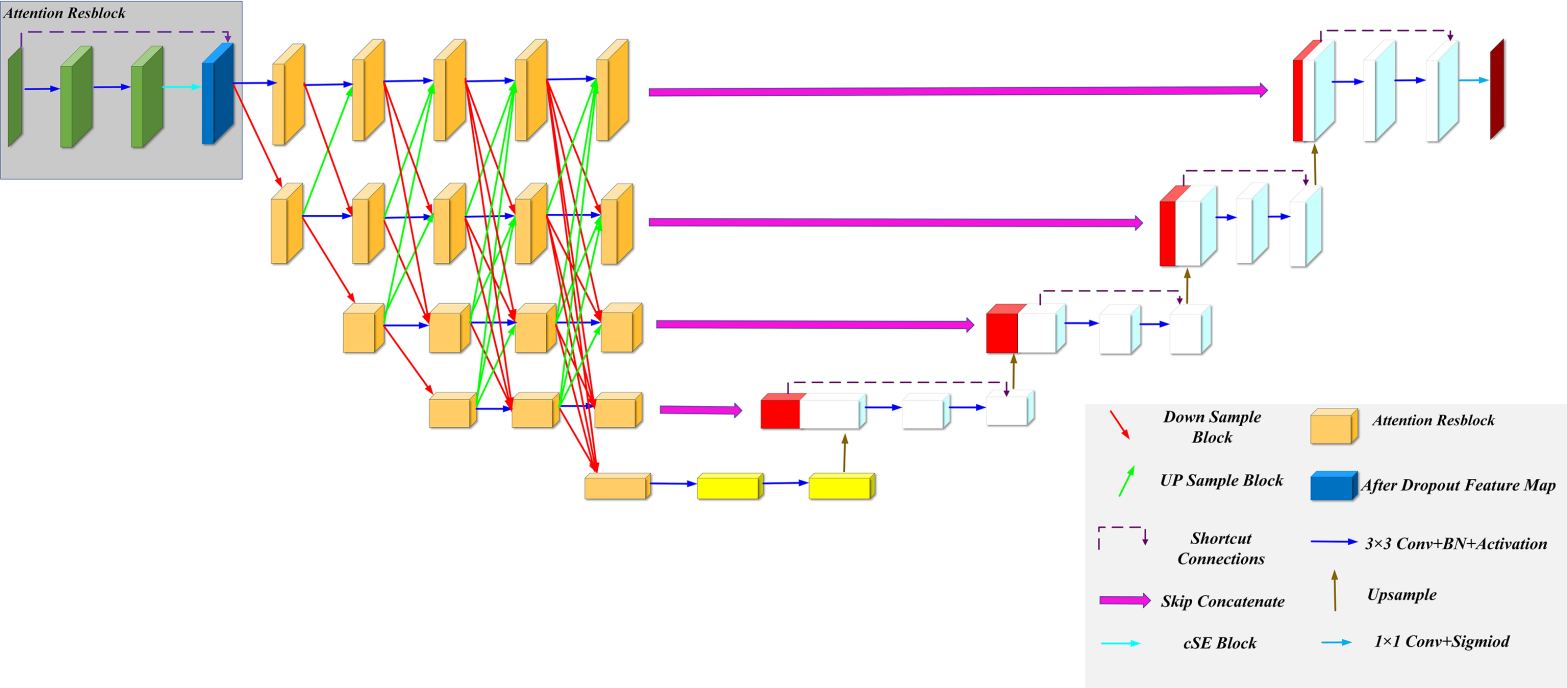
MSFA-U-Net structure.

**Figure 3:**
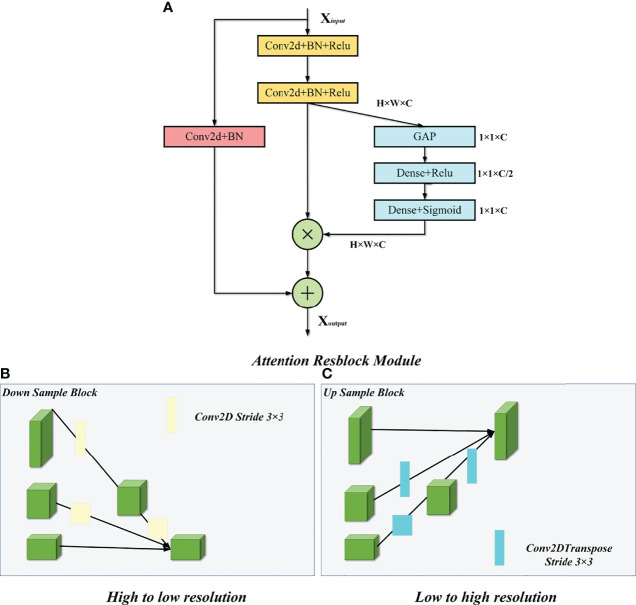
Attention Resblock Module and feature fusion of different scales. **(A)** Attention Resblock Module; blue cuboid = cSE module, red cuboid = Resblock module. **(B)** One or more stride convolutions (3 × 3) were used in the conversion from high to low resolution. **(C)** One or more transposed convolutions (3 × 3) were used in the conversion from low to high resolution.

We improved our model based on the U-Net and HRNet model architectures, called MSFA-U-Net. Main improvements were (a) replacing two feature extraction convolutions of different resolutions in the U-Net downsampling process with multiple convolution blocks in HRNet and feature fusion between different scales: and (b) introducing the cSE attention mechanism into each convolution block (**Figure 2**). In the downsampling process of the model, we connected a cSE module after extracting two 3 × 3 convolutional features and fused the input features with the post–scale operation features by means of a residual connection that consisted of a 1 × 1 2D convolution and a normalization layer (22) [batch normalization (BN)]. In the cSE module, we used a global average pooling (GAP) layer to convert a feature map from channel × height × width to channel × 1 × 1 and then used Dense to reduce the feature channel by half, which we achieved by activating the function Relu. Next, we restored the feature channel to normal size using Dense and activated it using the function Sigmoid. Finally, we obtained a calibrated feature map *via* channelwise multiplication. The schematic diagram of the residual connection and cSE module structure is shown in **Figure 3**. Residual connection can prevent gradient vanishing and gradient explosion during training (23). Moreover, the cSE module could effectively reflect relationships between different channels and assign different weights, enabling the model to focus on important features for accurate segmentation of the thyroid gland during the training process. The whole module is called an Attention Resblock (**Figures 2**, **3**). The traditional U-Net model uses the maximum pooling layer to perform downsampling and reduce the number of parameters; this method can lead to loss of information during feature extraction. Therefore, in this study we used stepped convolution for downsampling. Stride convolution can remove redundant information, thereby reducing the size of the feature map. Our model used multiple branches of different resolutions to extract features in parallel during the training process, and it performed feature fusion among different scales after each attention residual block to achieve strong semantic information and precise location during the training process. One or more transposed convolutions (3 × 3) were used in the conversion from low to high resolution, while one or more stride convolutions (3 × 3) were used in the conversion from high to low resolution (**Figure 3**). In the upsampling, the attention residual block replaced the two convolution operations in U-Net to avoid excessive parameters. Meanwhile, we added a dropout layer after each shortcut connection (parameter set to 0.2) to avoid a decrease in generalization caused by overfitting resulting from multiple feature fusions between different scales during the training process.

### Model Operating Environment and Parameters

We used TensorFlow software version 2.4.0 (Google Brain Team, 2015; Mountain View, CA, USA) and Keras software version 2.4.3 (Chollet, 2015) to build the model, and Python 3 (Van Rossum and Drake, 2009) to program it. In addition, we used a Windows 10 64-bit operating system (Microsoft Corp., Redmond, WA, USA) with the following hardware: central processing unit (CPU), Intel Core i9-10900 KF @ 3.70 GHz (Intel Corp., Santa Clara, CA, USA); graphics card, NVIDIA GTX3090 24 G (NVIDIA Corp., Santa Clara, CA, USA); and 128 GB memory. Model hyperparameters were selected from the best results according to the experimental conditions (**Table 1**). Batch Size represents the number of input images per iteration, Epoch represents the batch to be trained, Image Size represents the input size of the image, Learning Rate represents the initial learning rate using exponential decay, Decay steps indicate how many steps have been experienced for a learning rate decay, and Decay Rate indicates the learning rate decay coefficient.

**Table 1:** Network training parameters.

Model	Batch Size	Epoch	Image Size	Learning Rate	Decay Steps	Decay_Rate
U-Net	2	120	512 × 512	1e-5		
HRNet	2	120	512 × 512	8e-5	300	0.96
Attention U-Net	2	120	512 × 512	8e-4	300	0.96
MSFA-U-Net	2	120	512 × 512	2e-4	300	0.96

### Loss Function

Due to its small size, the thyroid gland occupies minimal space on a CT image. Therefore, use of the traditional cross-entropy loss function would leave the model more inclined to predict the background and thus unable to accurately identify the thyroid gland. Milletari et al. (24) proposed a loss function for sample imbalance in medical-image segmentation while researching V-Net-Dice loss function, which is based on DSC. It directly compares the overlap between the model prediction and real segmentation, thereby effectively solving the problem of serious thyroid imbalance. The Dice loss function is calculated according to formula (1.1) below:


(1.1) $$DL=1-2*\frac{\left|X\cap Y\right|+\varepsilon }{\left|X\right|+\left|Y\right|+\varepsilon },$$

where X represents the label matrix of the real thyroid gland, Y is the prediction matrix of the model predicting the thyroid gland, and ε represents a constant included to avoid division by zero.

### Evaluation Indices

We used the common indices of DSC, JSC, PPV, SE, and HD to further evaluate the generalization ability and segmentation accuracy of the model.

DSC (25) and JSC (26) were calculated according to formulas (1.2) and (1.3), respectively:


(1.2) $$DSC=2*\frac{\left|X\cap Y\right|}{\left|X\right|+\left|Y\right|},$$

(1.3) $$JSC=\frac{\left|X\cap Y\right|}{\left|X\cup Y\right|},$$

where X represents the standard segmentation map drawn by a radiologist, Y is the prediction image segmented by the neural-Network model, and | *X*∩*Y* | represents the overlap between the standard map drawn by the radiologist and the model-predicted image. The value range of DSC and JSC is 0–1; values closer to 1 indicate better predictive ability.

PPV (27) and SE (28) were calculated according to formulas (1.4) and (1.5), respectively:


(1.4) $$PPV=\frac{TP}{TP+FP},$$

(1.5) $$SE=\frac{TP}{TP+FN},$$

where TP represents the correctly predicted foreground target value, FP represents the incorrectly predicted foreground target value, and FN represents the incorrectly predicted background target value.

HD (29) was calculated according to formula (1.6):


(1.6) H(X,Y)=max(h(X,Y),h(Y,X)),

where:


$$h\left(X,Y\right)=\underset{x\in X}{\text{max}}\;\underset{y\in Y}{\text{min}}∥x-y∥,h\left(Y,X\right)=\underset{y\in Y}{\text{max}}\;\underset{x\in X}{\text{min}}∥y-x∥$$

Smaller values of HD indicate better predictive ability.

### Comparison Model Design

To prove the validity of the proposed MSFA-U-Net model, we selected three model architectures related to MSFA-U-Net and conducted comparative experiments:

1. U-Net (5): A U-shaped symmetrical structure composed of upsampling, downsampling, and skip connection. The skip connection effectively combines feature information among different resolutions and compensates for the loss of high-resolution features in the downsampling process. It could also output the feature map more accurately. U-Net is a widely used model in medicine.
2. HRNet (20): This model maintains high-resolution output during feature extraction. It has multiple parallel subnets with different resolutions to compress and extract features and to fuse features on multiple scales in order to obtain richer high-resolution features. In their original study, the authors used bilinear-interpolation upsampling. To better extract features for fusion, in this study we used transposed convolution to convert from low to high resolution.
3. Attention U-Net (30): This model introduces an attention-gating mechanism, which gives the information in the jump connection of the U-Net model different weights and enables it to pay more attention to the ROI.

## Experimental Results

### Qualitative Analysis of Results

**Figure 4:**
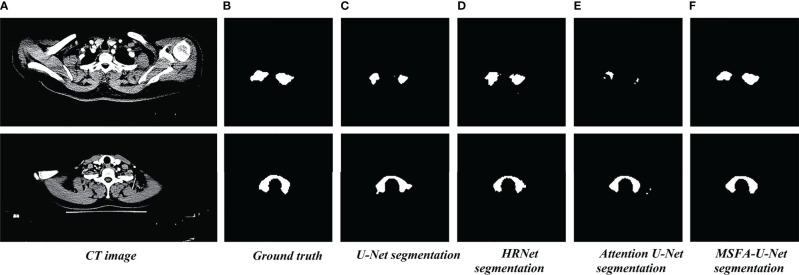
Thyroid gland segmentation of the four models on localized CT images for radiotherapy. **(A)** Standard image of the imported model (CT image). **(B)** Corresponding label image (Ground truth). **(C)** Thyroid segmented by U-Net. **(D)** Thyroid segmented by HRNet. **(E)** Thyroid segmented by Attention U-Net. **(F)** Thyroid segmented by MSFA-U-Net.

**Figure 5:**
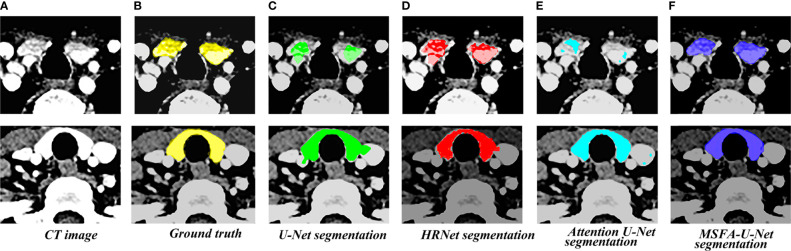
Thyroid coverage map of the four models on localized CT images for radiotherapy. **(A)** CT image. **(B)** Coverage map of thyroid of corresponding label image(Ground truth) on CT image. **(C)** Coverage map of thyroid segmented by U-Net on CT image **(D)** Coverage map of thyroid segmented by HRUet on CT image. **(E)** Coverage map of thyroid segmented by Attention U-Net on CT image. **(F)** Coverage map of thyroid segmented by MSFA-U-Net on CT image.

**Figure 4** shows the results of the thyroid gland segmentation of the four models in the test set. **Figure 5** shows the coverage map of the four models on the CT image of the radiotherapy location. Given the presence of many blood vessels and soft tissues with similar gray levels around the thyroid gland, oversegmentation and undersegmentation are expected in edge segmentation. As shown in **Figure 4**, some of the surrounding blood vessels and soft tissues were mistakenly segmented as part of the left lobe of the thyroid gland when the U-Net model segmented that lobe. Although HRNet and Attention U-Net decreased oversegmentation of the surrounding soft tissues and blood vessels, some noise points and uneven edges remained. However, the MSFA-U-Net architecture used in this study achieved smooth edges and decreased noise. Moreover, although we adjusted the window width and level and adopted adaptive contrast enhancement, some lesions at some levels of the thyroid might have been less obviously enhanced. At these levels, MSFA-U-Net exhibited more-robust thyroid segmentation than the other three models. In summary, compared with those other three models, MSFA-U-Net improved the performance of thyroid gland segmentation on localized CT images for radiotherapy.

### Quantitative Analysis of Results

**Table 2** compares the results for the four models in the test set of thyroid gland segmentation indices on localized CT images for radiotherapy. MSFA-U-Net had the best values for four evaluation indices: DSC, 0.90; JSC, 0.82; SE, 0.90; and HD, 2.39. Compared with the other three mainstream medical-image segmentation models, MSFA-U-Net greatly improved DSC (improvement range, 0.04–0.06), JSC (improvement range, 0.04–0.08), SE (improvement range, 0.04–0.11), and HD (improvement range, -0.21 to -0.06). On the PPV index, MSFA-U-Net was better than U-Net and worse than both HRNet and Attention U-Net; however, the HRNet and Attention U-Net models performed worse than the MSFA-U-Net model on the other evaluation indices.

**Table 2:** Assessment indices of the test set $\left(\bar{x}\pm s\right)$ .

	U-Net	HRNet	Attention U-Net	MSFA-U-Net
DSC	0.86 ± 0.10	0.84 ± 0.09	0.86 ± 0.15	**0.90 ± 0.09**
JSC	0.77 ± 0.13	0.74 ± 0.13	0.78 ± 0.16	**0.82 ± 0.11**
PPV	0.88 ± 0.12	0.93 ± 0.08	**0.95 ± 0.07**	0.91 ± 0.09
SE	0.86 ± 0.12	0.79 ± 0.13	0.81 ± 0.17	**0.90 ± 0.11**
HD	2.60 ± 0.57	2.59 ± 0.54	2.45 ± 0.69	**2.39 ± 0.54**

Bold, optimal value.

### Box Plot of Results

**Figure 6:**
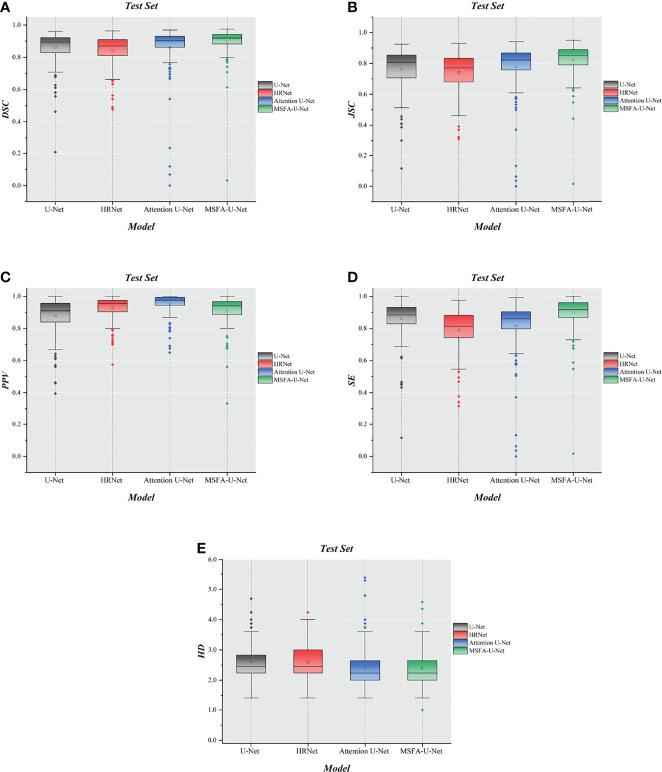
Box plot diagrams in the test set. **(A)** Box plot diagram of DSC in the test set. **(B)** Box plot diagram of JSC in the test set. **(C)** Box plot diagram of PPV in the test set. **(D)** Box plot diagram of SE in the test set. **(E)** Box plot diagram of HD in the test set.

To further evaluate the differences among the four models, we made box plots of the evaluation indices (**Figure 6**). The results showed that MSFA-U-Net had a smaller distance between the upper and lower quartiles than the other three models; it also had fewer outliers, and the outliers it did have were closer to the median. These findings indicated that MSFA-U-Net was better at segmenting the thyroid gland on localized CT images for radiotherapy than the other three models were; moreover, it ensured more-consistent segmentation results.

### Summary and Analysis of Results

Attention U-Net (which introduces gated attention) and HRNet did not show obvious advantages in thyroid gland segmentation on localized CT images for radiotherapy compared with U-Net; rather, they performed even worse on some of the evaluation indices. The reason might be that although the gated-attention mechanism can effectively segment the target category and location, it can also lead to an increase in false-positive model predictions for small-volume segmentation with variability in shape. HRNet performs multiple simple feature fusions; although it can effectively fuse features and obtain rich high-resolution features, it is also more likely to cause overfitting if training data is relatively scarce. Therefore, the phenomenon of integration leads to a decline in HRNet’s generalization ability. Clearly, the increases in model parameters and resource consumption might not necessarily improve results.

## Discussion

Radiotherapy is critical to comprehensive treatment of head and neck tumors and breast cancer. When radiotherapy plans are designed and implemented, accurate regulation of the radiation dose within the target area and limitation thereof to the surrounding OARs are important factors in the treatment plan’s evaluation. OARs must be precisely delineated to effectively limit the dose outside the target area and avoid side effects of radiation (31). The thyroid gland, as an OAR during treatment of head and neck tumors and breast cancer, must be protected during radiotherapy. Atlas-based Auto- contouring (ABAS) and deep learning contouring (DLC) are most widely used in present automatic delineation of OARs. Choi Ms et al. (32) compared the performance between ABAS and DLC in delineating breast cancer OARs and clinical target volume, which showed that DLC performed better than ABAS in the properties of most structures. Besides, L. V. van Dijk et al. (33). compared the performance between ABAS and DLC in automatically delineating head and neck OARs, which revealed that DLC had better performance than ABAS and DLC presented significantly better thyroid automatic delineation with DSC increasing 0.23 (0.83 VS 0.60). However, there still exists improvement space in automatic delineation of thyroid due to its small volume and complex shape, which necessitates developing a new DLC model to improve the accuracy of automatic delineation of the thyroid.

This study proposed a multi-scale fusion attention U-Net model to address the problem of thyroid gland segmentation on localized CT images for radiotherapy. The innovation of this algorithm lay in the addition of multiple parallel channels on the basis of the traditional U-Net model. It fully integrated feature information between different resolutions, thereby avoiding single-resolution information in the U-Net downsampling process. In addition, our study also introduced the cSE attention mechanism, which inclined the model to the ROI during the training process. The experimental results showed that, compared with similar representative segmentation algorithms, the proposed model improved both qualitative and quantitative results to a certain extent and had better robustness and generalization. The image segmentation graphs revealed that MSFA-U-Net effectively reduced oversegmentation and undersegmentation and achieved smoother edges. It is believed in the relevant articles that DSC > 0.70 indicates acceptable agreement (34, 35). All models used in this study reached this threshold with the DSC value of MSFA-U-Net reaching 0.90, indicating that this model could effectively segment the thyroid gland on localized CT images for radiotherapy. The box plot diagrams demonstrated that MSFA-U-Net yielded good quantitative results: the upper- and lower-quartile gaps and outliers of most evaluation indicators were reduced, indicating that the model achieved consistent segmentation of the different layers of the thyroid gland and could segment the gland effectively.

Furthermore, some automatic delineation performance of the thyroid conducted by other researchers were compared with ours. L. V. van Dijk et al. (33) adopted deep learning contouring to improve automatic delineation for head and neck OARs. In their study, automatic delineation of 693 patients were performed with DSC and HD being 0.83 ± 0.08 and 3.6 ± 3.0 mm for DLC. In our study, the DSC and HD for the proposed model, MSFA-U-Net, were 0.90 ± 0.09, and 2.39 ± 0.54 respectively. Yang et al. (36) used a self-adaptive Unet network to segment OARs on the CT images of 149 nasopharyngeal carcinoma patients and obtained thyroid segmentation with DSC being 0.83 ± 0.03 and HD being 4.5 ± 1.3. Compared with their model, our proposed model, MSFA-U-Net, increased DSC by 0.07 and reduced HD by 2.11, indicating that MSFA-U-Net had certain superiority in segmenting the thyroid gland on localized CT images for radiotherapy. Zhong et al. (37) proposed Boosting-based Cascaded Convolutional Neural Network to segment the head and neck OARs. In their model, DSC was above 92.29% and HD was 2.64 for the thyroid. Our proposed model produced a lower DSC, but a better HD evaluation. Cascaded and boosting were adopted in their model, which promoted its performance than single-models but meanwhile increased its complexity. When we take model complexity and segmentation performance into consideration, we find that our model still has its advantages.

However, the algorithm proposed in this study had some limitations. First, certain MSFA-U-Net evaluation indices have not yet reached optimal results. Second, the introduction of numerous feature fusions among different scales increased the number of model parameters. Third, although Dice loss function could effectively solve the problem of class imbalance, its gradient characteristics could cause the model to oscillate during the training process. In future research, we will explore how to reduce the parameter count of the model while further optimizing the model by using different loss functions.

## Conclusion

In summary, the MSFA-U-Net model enabled radiotherapy physicians to automatically delineate the thyroid gland on localized CT images for radiotherapy. Our results showed that the model could be applied in clinical work: compared with the three commonly used models in medicine, MSFA-U-Net could delineate the thyroid gland more accurately.

## Data Availability Statement

The raw data supporting the conclusions of this article will be made available by the authors, without undue reservation.

## Author Contributions

All authors were involved in the conception of this study, design, and implementation. XW was a major contributor in designing the models, drawing the figures and writing the manuscript and was also involved in delineating some label images. YY performed the checking and proofing of the manuscript and the data apart from guidance to the writing of the manuscript. BZ, MY, JL, MS, LM, and CS were major contributors in data collection, delineation of most of the label images and output of data. All authors contributed to the article and approved the submitted version.

## Funding

This study has received funding by Climbing Foundation of National Cancer Center (NCC201925B03); Funding unit: National Cancer Center; Funder: YY: Director, Department of Radiotherapy, Yunnan Cancer Hospital.

## Conflict of Interest

The authors declare that the research was conducted in the absence of any commercial or financial relationships that could be construed as a potential conflict of interest.

## Publisher’s Note

All claims expressed in this article are solely those of the authors and do not necessarily represent those of their affiliated organizations, or those of the publisher, the editors and the reviewers. Any product that may be evaluated in this article, or claim that may be made by its manufacturer, is not guaranteed or endorsed by the publisher.

## Abbreviations

CT, computed tomography; MSFA-U-Net, Multi-scale Fusion Attention U-Net; DSC, Dice similarity coefficient; JSC, Jaccard similarity coefficient; PPV, positive predictive value; SE, sensitivity; HD, Hausdorff distance; HRNet, High-Resolution net; OARs, organs at risk; AI, artificial intelligence; DL, deep learning; MRI, magnetic resonance imaging; MALF, multi-atlas label fusion; RF, random forest 3D, three-dimensional; 3D-CRT, three-dimensional conformal radiotherapy; ROI, region of interest; cSE, Spatial Squeeze and Channel Excitation Block; BN, batch normalization; GAP, global average pooling; DLC, deep learning contouring.
